# Library Screening for Synergistic Combinations of FDA-Approved Drugs and Metabolites with Vancomycin against VanA-Type Vancomycin-Resistant Enterococcus faecium

**DOI:** 10.1128/spectrum.01412-22

**Published:** 2022-08-15

**Authors:** Shivani Gargvanshi, William G. Gutheil

**Affiliations:** a Division of Pharmacology and Pharmaceutical Sciences, School of Pharmacy, University of Missouri-Kansas City, Kansas City, Missouri, USA; Indian Institute of Science Bangalore

**Keywords:** library screening, drug repurposing, synergy screening, *Enterococcus faecium*, microsome, metabolism, antibiotic drug resistance, VRE, antimicrobial resistance, cytochrome P450, drug metabolism, vancomycin resistance

## Abstract

Antimicrobial resistance is a major public health threat, and there is an urgent need for new strategies to address this issue. In a recent study, a library screening strategy was developed in which an FDA-approved drug library was screened against methicillin-resistant Staphylococcus aureus (MRSA) in both its original (unmetabolized [UM]) and its human liver microsome metabolized (postmetabolized [PM]) forms and in the absence and presence of a resistant-to antibiotic. This allows the identification of agents with active metabolites and agents that can act synergistically with the resistant-to antibiotic. In this study, this strategy is applied to VanA-type vancomycin-resistant Enterococcus faecium (VREfm) in the absence and presence of vancomycin. Thirteen drugs with minimum MICs that were ≤12.5 μM under any tested condition (UM/PM vs. −/+vancomycin) were identified. Seven of these appeared to act synergistically with vancomycin, and follow-up checkerboard analyses confirmed synergy (∑FICmin ≤0.5) for six of these. Ultimately four rifamycins, two pleuromutilins, mupirocin, and linezolid were confirmed as synergistic. The most synergistic agent was rifabutin (∑FICmin = 0.19). Linezolid, a protein biosynthesis inhibitor, demonstrated relatively weak synergy (∑FICmin = 0.5). Only mupirocin showed significantly improved activity after microsomal metabolism, indicative of a more active metabolite, but efforts to identify an active metabolite were unsuccessful. Spectra of activity of several hits and related agents were also determined. Gemcitabine showed activity against a number vancomycin-resistant E. faecium and E. faecalis strains, but this activity was substantially weaker than previously observed in MRSA.

**IMPORTANCE** Resistance to currently used antibiotics poses a serious threat to public health. This study reports a complete screen of 1,000 FDA-approved drugs and their metabolites against vancomycin-resistant Enterococcus faecium (VREfm) in both the absence and presence of vancomycin. This identified potentially synergistic combinations of FDA-approved drugs with vancomycin, and a number of these were confirmed in follow-up checkerboard assays. Among intrinsically active FDA-approved drugs, gemcitabine was identified as having activity against a panel of VRE strains.

## INTRODUCTION

Pathogenic bacteria are becoming increasingly drug resistant, with some now virtually untreatable (1–4). There has concurrently been a lack of new antibacterial agents identified over the last 30 years to counter this threat (4–6). *Enterococcus* spp. are Gram-positive commensal bacteria of the intestine in humans and animals that can cause problematic infections of the gasterointestinal (GI) tract and soft tissues (7–10). Vancomycin (Vm) is one of the most important agents for the treatment of G+ bacterial infections resistant to most other antibacterial agents, including Vm-sensitive enterococcal (VSE) and methicillin-resistant Staphylococcus aureus (MRSA) infections (11–13). The emergence and spread of Vm-resistant *Enterococcus* spp. (VRE) are serious public health issues given the lack of alternatives for these organisms and their increasing resistance to currently used agents (8, 9, 11, 14). VRE is a WHO high priority pathogen for new agent development (15).

Chemical compound library screening is a core approach for the discovery of new bioactive agents, including for antibacterial activity. However, large untargeted (whole cell) and targeted (individual protein) library screening efforts have given overall disappointing results (16, 17). An alternative to large library screens are smaller scale efforts with high value libraries such as FDA-approved drug library screening, which has become a popular strategy for “drug repurposing” (18). This strategy can reveal novel new activities of FDA-approved drugs, which provides a greatly shortened path to clinical application. Another strategy to counter antimicrobial resistance is to identify agents that can act synergistically with or restore the activity of another antibiotic (5, 19, 20). Many drugs are also known to have active metabolites (21, 22). Comparative screening of the unmetabolized (UM) and postmetabolized (PM) libraries allows agents with increased antibacterial activity to be identified for deconvolution and active metabolite identification, as recently demonstrated using a human liver microsome-metabolized FDA-approved drug library (23).

In a prior study we have demonstrated an approach in which FDA-approved drug library screening is performed against MRSA with both FDA-approved drugs and their metabolites and simultaneously in the absence and presence of a resistant-to antibiotic (23). This allows intrinsically active agents, agents with active metabolites, and agents synergistic with the resistant-to antibiotic to be identified in a single screen. In this study, this approach was used to screen an FDA-approved library in its original (unmetabolized [UM]) and human microsome metabolized (postmetabolized [PM]) versions against vancomycin-resistant Enterococcus faecium (VREfm) in the absence and presence of sub-MIC levels of vancomycin (−/+Vm) (2 × 2 library screening design).

## RESULTS AND DISCUSSION

While there has been some progress in the development of new antibacterial agents to combat the emergence and spread of antimicrobial resistance (AMR) in pathogenic bacteria (24), there has been a lack of new agents without established resistance mechanisms (5, 25–28). New agents of novel mechanism are urgently needed. Another approach to combating AMR is the identification of effective synergistic agent combinations, particularly from the repertoire of existing antibacterial agents (20, 29–31). Such synergistic agent combinations have enhanced activity against targeted organisms and can also reduce the emergence and spread of resistance. Both new agent identification and the identification of new synergistic agent combinations are potential pathways in addressing the problem of AMR. The development and demonstration of effective approaches that can perform both types of screens simultaneously would be useful for efforts to address AMR.

In a prior study, we demonstrated in MRSA how a two-dimensional screening strategy comparing an unmetabolized (UM) versus postmetabolized (PM) FDA library screen combined with a −/+ resistant-to antibiotic screen could enhance the ability to identify new agents and new synergistic combinations (23, 32). This study identified gemcitabine as having generally good anti-MRSA activity, identified strong synergy between cefoxitin and floxuridine against MRSA, and also identified capecitabine as having several anti-MRSA metabolites. Focusing this approach on an FDA-approved drug library offers the potential of identifying novel antibacterial activities in FDA-approved drugs and FDA-approved drug metabolites, and identifying novel synergistic drug combinations, all features demonstrated previously (23). The rationale for this strategy is to perform screening replication under somewhat different but informative conditions. Screening of the PM library allows FDA-approved drugs with active metabolites to be identified, and screening in the absence and presence of a resistant-to antibiotic allows agents acting synergistically with the resistant-to antibiotic to be identified.

In this study, this effort was extended to a vancomycin-resistant Enterococcus faecium strain in the absence and presence of vancomycin. Library screens were performed at 50 μM of the library compounds and in the absence or presence of 16 μg/mL vancomycin (1/8× MIC). This vancomycin level was selected as a compromise between a higher 1/4× MIC concentration of 32 μg/mL and a lower more clinically relevant concentration that would be unlikely to reveal synergies with vancomycin. Following library screening a merged hit list was made, in which any compound that gave a hit (was active) under any of the four screening conditions (UM − Vm, UM + Vm, PM − Vm, and PM + Vm) was included in the list. MICs for all the compounds in this pooled hit list were then determined for UM library compounds in both the absence and presence of 16 μg/mL vancomycin and for PM library compounds in both the absence and presence of 16 μg/mL vancomycin to give a preliminary table of MICs. MICs for compounds which gave a minimum MIC over all four tested conditions of ≤25 μM were then determined at least in triplicate. The results for agents with an MIC_min ≤12.5 (under all 4 test conditions) are summarized in Table 1. A complete list of all active and inactive agents is provided as Tables S2 and S3, respectively.

**TABLE 1:** FDA library anti-VREfm (clinical isolate) hit MICs (Min_MIC ≤12.5 μM)

	UM MICs (μM)		PM MICs (μM)						
Compound	−Vm	+Vm	−Vm	+Vm	Min_MIC	L2_(UM/PM)_^*a*^	AL2_(UM/PM)_^*b*^	L2_(UM−/+Vm)_^*c*^	AL2_(−/+Vm)_^*d*^
Rifampin	0.10	2.4 × 10^−2^	12.5	12.5	2.4 × 10^−2^	−7	−8	**2** ^*e*^	1
Rifapentine	0.20	2.4 × 10^−2^	25	12.5	2.4 × 10^−2^	−7	−8	**3**	**2** ^*e*^
Retapamulin	0.20	4.9 × 10^−2^	3.1	1.6	4.9 × 10^−2^	−4	−4.5	**2**	1.5
Rifaximin	0.39	4.9 × 10^−2^	50	50	4.9 × 10^−2^	−7	−8.5	**3**	1.5
Rifabutin	0.20	9.8 × 10^−2^	25	6.25	9.8 × 10^−2^	−7	−6.5	1	1.5
Valnemulin	0.39	9.8 × 10^−2^	0.20	9.8 × 10^−2^	9.8 × 10^−2^	1	0.5	**2**	1.5
Gemcitabine	0.78	0.20	25	25	0.20	−5	−6	**2**	1
Mupirocin	3.1	0.78	0.78	0.39	0.39	**2** ^*e*^	1.5	**2**	1.5
Closantel	1.6	1.6	12.5	12.5	1.6	−3	−3	0	0
Novobiocin	3.1	1.6	3.1	3.1	1.6	0	−0.5	1	0.5
Fidaxomicin	12.5	6.25	100	100	6.25	−3	−3.5	1	0.5
Florfenicol	25	25	12.5	6.25	6.25	1	1.5	0	0.5
Linezolid	3.1	1.6	6.25	6.25	6.25	−1	−1.5	1	0.5

a $\text{L}2_{\left(\text{UM}/\text{PM}-\text{Vm}\right)}={\text{log}}_{2}(\frac{{\text{MIC}}_{\text{UM}-\text{Vm}}}{{\text{MIC}}_{\text{PM}-\text{Vm}}}).$

b $\text{AL}2_{\left(\text{UM}/\text{PM}\right)}=\text{Avg}({\text{log}}_{2}\left(\frac{{\text{MIC}}_{\text{UM}-\text{Vm}}}{{\text{MIC}}_{\text{PM}-\text{Vm}}}\right),{\text{ log}}_{2}\left(\frac{{\text{MIC}}_{\text{UM}+\text{Vm}}}{{\text{MIC}}_{\text{PM}+\text{Vm}}}\right)).$

c $\text{L}2_{\left(\text{UM}-/+\text{Vm}\right)}={\text{log}}_{2}(\frac{{\text{MIC}}_{\text{UM}-\text{Vm}}}{{\text{MIC}}_{\text{UM}+\text{Vm}}}).$

d $\text{AL}2_{\left(-/+\text{Vm}\right)}=\text{Avg}({\text{log}}_{2}\left(\frac{{\text{MIC}}_{\text{UM}-\text{Vm}}}{{\text{MIC}}_{\text{UM}+\text{Vm}}}\right),{\text{ log}}_{2}\left(\frac{{\text{MIC}}_{\text{PM}-\text{Vm}}}{{\text{MIC}}_{\text{PM}+\text{Vm}}}\right)).$

e Values ≥2 are indicative of a significant decrease in MIC and a significant increase in potency and are in bold. For UM/PM ratios, this is indicative of a possible active metabolite, and for −/+Vm ratios this is indicative of a possible synergistic interaction between the drug and vancomycin.

Several assessments and comparisons are possible using the Table 1 MIC data (23). The first is to assess for interesting intrinsically active agents as revealed by examining the UM − Vm data in Table 1. Most of the agents listed in the UM − Vm column are well-known antibacterial agents with the exception of gemcitabine and closantel. Closantel is a veterinary antiparasitic drug that has previously been identified as having anti-MRSA and anti-VRE activity (33–36). Gemcitabine has also previously been identified as having anti-MRSA activity (37, 38), including in our own efforts (32), but its anti-VRE activity appears previously unrecognized. The spectrum of activity of several of the better agents from Table 1 was determined against a panel of VRE isolates (both VREfm and E. faecalis [VREfa]), in which gemcitabine demonstrated activity against all tested VRE strains with a median MIC of 0.78 μM (Table 2). This is a higher median MIC than against a panel of MRSA strains where the median MIC was 0.049 μM. These results indicate that gemcitabine and similar agents may have some potential for further development as anti-VRE and anti-MRSA agents. Several other nucleoside analogs were also tested for activity against these VRE strains (Table 3), but these did not exhibit the same broad anti-VRE activity as they did against MRSA (23).

**TABLE 2:** Spectra of activity (UM − Vm) against VRE^*a*^

Compound	VREfm (clinical)^*b*^^,^^*c*^	VREfm (BAA-2317)	VREfm (BAA-2318)	VREfm (BAA-2365)	VREfa (49532)	VREfa (49533)	VREfa (51575)	VREfa (700802)
Rifampin	0.10	NA^*d*^	0.39	1.6	6.25	6.25	6.25	6.25
Retapamulin	0.20	9.8 × 10^−2^	9.8 × 10^−2^	NA	NA	NA	NA	NA
Rifabutin	0.20	NA	0.78	3.1	25	25	25	25
Rifapentine	0.20	NA	1.6	3.1	6.25	6.25	6.25	12.5
Rifaximin	0.39	50	25	1.6	1.6	1.6	3.1	1.6
Valnemulin	0.39	0.39	0.39	NA	NA	NA	NA	NA
Gemcitabine	0.78	0.39	2.4 × 10^−2^	0.78	1.6	1.6	0.78	3.1

a MICs are in μM.

b Values from Table 1.

c The VREfm used in this study was a clinical isolate from the University of Missouri-Kansas City School of Medicine. For other strains, the American Type Culture Collection (ATCC) catalog numbers are given in parentheses.

d NA, not active at 50 μM, the highest concentration used in these MIC determinations.

**TABLE 3:** Spectra of activity of doxifluridine, floxuridine, and 5′-fluorouracil against VRE^*a*^

Compound	VREfm (clinical)	VREfm (BAA-2317)^*b*^	VREfm (BAA-2318)	VREfa (BAA-2365)	VREfa (49532)	VREfa (49533)	VREfa (51575)	VREfa (700802)
DFUR	NA^*c*^	NA	NA	NA	NA	NA	50	NA
Floxuridine	NA	NA	NA	3.1	25	50	6.25	50
5-Fluorouracil	NA	NA	NA	3.1	25	25	12.5	50

a MICs are in μM. DFUR, doxifluridine.

b American Type Culture Collection (ATCC) catalog numbers are in parentheses.

c NA, not active at 50 μM, the highest concentration used in these MIC determinations.

The second assessment is to compare Table 1 MICs to identify agents with increased activity after metabolism, indicative of more active metabolites. Nearly all drugs are transformed into at least one metabolite, and such metabolites frequently have distinct biological activities (21, 22). Our prior study demonstrated the potential of a UM versus PM library screen to identify active drug metabolites (23). The effect of microsomal metabolism on compound activity against VREfm is highlighted in Table 1 in the L2_(UM/PM−Vm)_ value. L2_(UM/PM−Vm)_ is the log base 2 value of the ratio between the MIC_UM−Vm_ and MIC_PM−Vm_ values, as defined in Table 1. The AL2_(UM/PM)_ represents the average of log 2 value of MIC_UM−Vm_/MIC_PM−Vm_ and log 2 value of MIC_UM+Vm_/MIC_PM+Vm_ as defined in Table 1 (23). These values place the fold change in MIC values between various treatments on a log scale. Values of L2_(UM/PM−Vm)_ ≥2 (i.e., 4-fold reduction in MIC) are in bold and indicate substantially increased potency (lower MIC) after metabolism. Only mupirocin met this standard after metabolism (Table 1), suggesting the possibility of an active metabolite. However, we were unable to identify an active metabolite. While library metabolism was useful in identifying novel anti-MRSA compounds (23), it was not successful when applied to VREfm.

**FIG 1:**
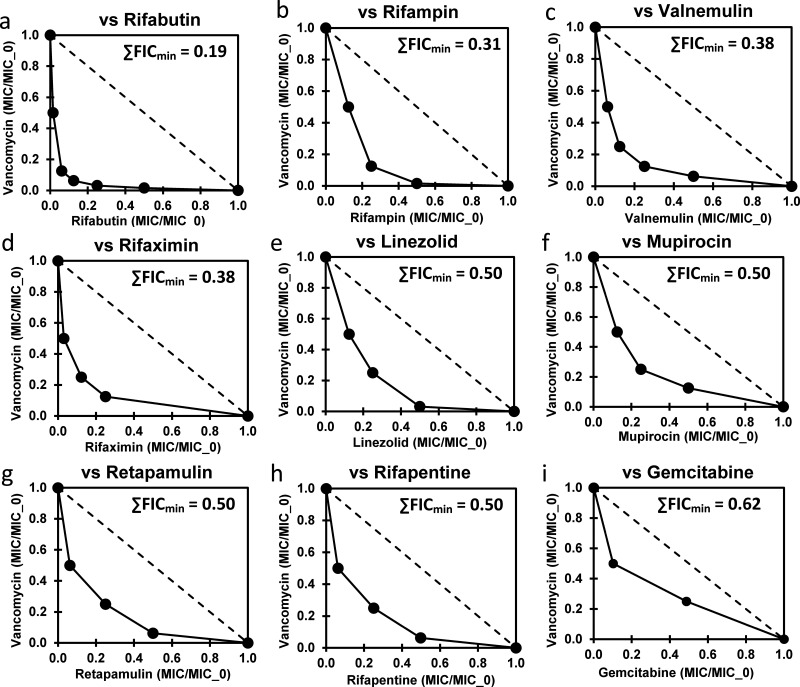
(a to i) Isobolograms from checkerboard assay results for combinations of vancomycin with potentially synergistic agents against VREfm (clinical isolate). The dashed line in the isobolograms is for the no interaction (additive MICs) curve. MICs for other agents alone as given in Table 1.

The third assessment from Table 1 data is comparisons to reveal possible synergistic agent combinations with vancomycin (Table 1; L2_(UM−/+Vm)_ and AL2_(−/+Vm)_ columns). This identified seven potential agents with vancomycin synergy: rifampin, rifapentine, retapamulin, rifaximin, valnemulin, gemcitabine, and mupirocin. Checkerboard assays revealed significant synergies (∑FICmin ≤0.5) for six of these, but not for gemcitabine (Fig. 1). Since several of these were rifamycins, synergy for rifabutin was also tested for and confirmed (Fig. 1a). The observation of synergy of these rifamycins, RNA biosynthesis inhibitors, with vancomycin is interesting. Rifampicin synergy with vancomycin has been previously observed with several VRE strains and with no synergy for vancomycin-sensitive enterococcal strains (39). Both pleuromutilin (retapamulin and valnemulin) protein biosynthesis inhibitors also demonstrated synergy with vancomycin (Fig. 1g and c, respectively). A checkerboard assay was then performed between linezolid and vancomycin, and this was also confirmed as a synergistic combination (Fig. 1e). Mupirocin, an Ile-tRNA biosynthesis inhibitor that induces (p)ppGpp biosynthesis (stringent response) (40, 41), also demonstrated modest synergy (Fig. 1f). These observations demonstrate that direct RNA biosynthesis inhibitors (rifamycins), indirect RNA biosynthesis inhibitors (mupirocin), and protein biosynthesis inhibitors (pleuromutilins and linezolid) can all act synergistically with vancomycin on VREfm. Vancomycin resistance in this strain of VREfm is inducible (42), and the ability of these agents to block RNA and protein biosynthesis likely blocks the ability of this VREfm strain to express high level vancomycin resistance. Rifabutin was the most synergistic of these agents for reasons which are currently unknown. The report of rifampin-vancomycin synergy in VRE strains, but not in VSE strains (39), also supports this conclusion.

The overall goal of this effort was to further demonstrate the utility of enhanced library screening approaches in which replicate library screens are performed with variation between the replicates. The basic screen (UM − Vm) identified both closantel and gemcitabine as nontypical anti-VRE agents. The spectrum of activity of closantel against a number of VRE strains has previously been reported (36). Gemcitabine was demonstrated in this study (Table 2) to also have activity against a number of VRE strains. The molecular target of gemcitabine is unknown, but further studies of gemcitabine and homologs seem justified from these observations. No agents with identifiable active metabolites were discovered in this screen, in contrast to the identification of active capecitabine metabolites in MRSA (23). Screening for synergistic combinations with vancomycin revealed a number of synergistic agents (Fig. 1). These were all either RNA or protein biosynthesis inhibitors, suggesting a common mechanistic basis for these synergies. Some of the agents and agent combinations identified in this effort may be suitable candidates for further *in vitro* and *in vivo* studies, and ultimately clinical application.

## MATERIALS AND METHODS

### General.

The reagents and materials used in this study were as described previously (23). Bacterial strains were obtained from American Type Culture Collection (ATCC; Manasas, VA) and the Biodefense and Emerging Infections Research Resources Repository (BEI; Manasas, VA). The bacterial strain used for library screening was a VanA-type vancomycin-resistant Enterococcus faecium (VREfm) clinical strain we have used in other studies (42). Other bacterial strains used in this study were as indicated in the appropriate tables. UM and PM working library plates including control antibiotics and control microsomal metabolism substrates were prepared as described previously (23) at 0.5 mM. VRE growth medium consisted of brain heart infusion (37.5 g/L), hemin (10 mg/L), and NAD+ (10 mg/L).

### UM/PM versus −/+Vm library screen against VREfm.

Four sets of library screening plates were prepared for the following screens; UM − Vm, UM + Vm, PM − Vm, and PM + Vm. Two sets of UM plates and two sets of PM plates were first prepared from working library samples (2 μL at 0.5 mM working library samples per well) in 384-well Corning microtiter plates (catalog no. 3680) using a Biomek 3000 liquid handing workstation. Plates were frozen at −80°C and dried under strong vacuum (<50 μm Hg) in a Genevac Quatro centrifugal concentrator. To each well in each set was added 20 μL of VRE growth medium containing 4,000 CFU VRE and containing either no Vm for −Vm screens or +16 μg mL^−1 ^Vm (1/8× MIC) for +Vm screens. These additions were performed using an Integra Viaflo Assist automated multichannel pipette (Hudson, NH) in a Labconco (Kansas City, MO) BSL-2 biosafety cabinet. Plates were incubated for 48 h at 35°C. Fresh VRE growth medium (10 μL) was added to the wells of these four sets of plates, followed by incubation for 2 h at 35°C to restart active cell growth. To the wells of these plates was then added 10 μL of 100 μg mL^−1^ resazurin (sodium salt) (43–45). The plates were incubated for another 2 h at 35°C, and the A_610_ – A_450_ absorbance difference (Promega Technical Bulletin TB317) was measured in a Molecular Devices SpectraMax M5 multimode microplate reader (San Jose, CA).

### Hit picking and MIC determination.

Library screening data were processed and analyzed using homemade Matlab scripts (The Mathworks, Natick, MA). Based on the values for known active and inactive antibacterial agent controls, a cut-off value between active and inactive compounds was selected and lists of active wells in each screening set (UM − Vm, UM + Vm, PM − Vm, and PM + Vm) were generated. These lists were merged to give a pooled hit list. Rows were added to this pooled hit list to include known active and inactive antibiotics containing wells as controls. MICs were determined by hit picking 2-μL samples from both UM and PM working plates (two sets from each) into the first columns of 384-well plates (four sets total, for UM − Vm, UM + Vm, PM − Vm, and PM + Vm MIC determinations). These samples were then serially diluted in steps of two across the plates with DMSO using an Integra Viaflo Assist automated multichannel pipettor. The last column was left blank (DMSO only). These plates were frozen at −80°C and dried under strong vacuum as described above. To each well in each set was added 20 μL VRE growth medium containing 4000 CFU VREfm and containing either no Vm for −Vm MICs or 16 μg mL^−1 ^Vm for +Vm MICs. (This provided MIC plates with 50 μM as the highest test agent concentration.) Plates were incubated for 48 h at 35°C. Fresh VRE growth medium (10 μL) was added to the wells of these four sets of plates, followed by incubation for 2 h at 35°C to restart active cell growth. To the wells of these plates was then added 10 μL of 100 μg mL^−1^ resazurin (43–45). The plates were incubated for another 2 h at 35°C, and the A_610_ – A_450_ absorbance difference was measured as described above. MICs were determined using a cutoff midway between known active and inactive samples. All MICs were determined at least in triplicate and at least in quadruplicate for MIC_min ≤ 25 μM to ensure reproducibility.

### Spectrum-of-activity of VRE hits.

MICs were determined against a panel of VREfm and VREfa strains (Table 2). Plates were prepared by serial dilution of compounds in DMSO across 384-well plates and drying under high vacuum as described above.

### Checkerboard assays to confirm synergy.

Checkerboard assays (29) were performed to confirm synergy for prospective synergistic agents. Checkerboard assays were performed in 96-well plates from DMSO compound stocks using serial dilutions in steps of two in both dimensions in DMSO, and plates were then dried under vacuum as described above. To these plates was added 100 μL VRE growth medium containing 4,000 CFU VRE to each well, and plates were incubated for 48 h at 35°C. Fresh VRE growth medium (50 μL) was added, plates were incubated at 35°C for 2 h, 50 μL of 100 μg mL^−1^ resazurin was added, plates were incubated an additional 2 h, and the resazurin absorbance difference was measured as described above. All checkerboard assays were performed at least in triplicate and averaged.
