# Supplementary material for: Library Screening for Synergistic Combinations of FDA-Approved Drugs and Metabolites with Vancomycin against VanA-Type Vancomycin-Resistant Enterococcus faecium
Source: Microbiol Spectr. 2022 Aug 15;10(5):e01412-22. doi: 10.1128/spectrum.01412-22 (PMC9603392; doi:10.1128/spectrum.01412-22)
Supplement: Supplemental file 1 — Supplemental material. Download spectrum.01412-22-s0001.pdf, PDF file, 0.1 MB [file spectrum.01412-22-s0001.pdf]

Supplementary Information

Library Screening for Synergistic Combinations of FDA Approved Drugs and Metabolites with Vancomycin against VanA-type Vancomycin-Resistant *Enterococcus faecium*

Shivani Gargvanshi and William G. Gutheil*

Division of Pharmacology and Pharmaceutical Sciences, School of Pharmacy, University of Missouri-Kansas City, 2464 Charlotte Street, Kansas City, MO 64108

*Corresponding author: William G. Gutheil, Division of Pharmacology and Pharmaceutical Sciences, School of Pharmacy, University of Missouri Kansas City, 2464 Charlotte Street., Kansas City, MO, 64108, USA, Tel.: (816) 235-2424; Fax: (816) 235-5779; E- mail: gutheilw@umkc.edu

Running title: Enhanced FDA vs VREfm screening.

Keywords: Library screening; Drug repurposing; *Enterococcus faecium*; Microsome; P450; Metabolism; Chemical Diversity; Synergy; Antibiotic drug resistance; VRE.

Contents

I. **Supplementary Table S1** List of actives (validated min_MIC < 100 µM) from library screening against VREfm (clinical) (UM/PM vs -/+ 16 µg/mL vancomycin) ranked by lowest minimum MIC..

Supplementary Table 2. List of inactives from library screening against MRSA (ATCC 43300).

Table S1. List of actives (validated min_MIC < 100 µM) from library screening against VREfm (clinical) (UM/PM vs -/+ 16 µg/mL vancomycin) ranked by lowest minimum MIC. NA = no activity.

		UM MICs (µM)		PM MICs (µM)		
Compound	CAS_Number	-Vm	+Vm	-Vm	+Vm	Min_MIC
Retapamulin	224452-66-8	0.10	4.9E-2	3.1	1.6	4.9E-2
Valnemulin	133868-46-9	0.39	9.8E-2	0.20	9.8E-2	9.8E-2
Mupirocin	12650-69-0	3.1	0.78	0.78	0.39	0.39
Rifapentine	61379-65-5	0.2	2.4E-2	25	12.5	2.4E-2
Novobiocin	1476-53-5	3.1	1.6	3.1	3.1	1.60
Fidaxomicin	873857-62-6	12.5	6.25	NA	NA	6.25
Closantel	57808-65-8	1.6	1.6	12.5	12.5	1.60
Florfenicol	73231-34-2	25	25	12.5	6.25	6.25
Cetrimonium Bromide	57-09-0	12.5	12.5	NA	NA	12.5
Linezolid	165800-03-3	3.1	1.6	6.25	6.25	1.60
Rifampin	13292-46-1	0.10	2.4E-2	12.5	12.5	2.4E-2
Rifaximin	80621-81-4	0.39	4.9E-2	50	50	4.9E-2
Gemcitabine	95058-81-4	0.78	0.20	25	25	0.20
Rifabutin	72559-06-9	0.20	9.8E-2	25	6.25	9.8E-2
Cetylpyridinium Chloride	123-03-5	50	25	NA	NA	25
Daunorubicin	23541-50-6	50	25	NA	NA	25
Methacycline	3963-95-9	50	25	NA	NA	25
Sitafloxacin	163253-35-8	25	25	NA	NA	25
Thiamphenicol	15318-45-3	50	50	25	25	25
Balofloxacin	127294-70-6	NA	NA	50	50	50
Benzethonium chloride	121-54-0	50	50	NA	NA	50
Candesartan cilexetil	145040-37-5	NA	50	NA	NA	50
Clomifene citrate (Serophene)	50-41-9	NA	50	NA	NA	50
Domiphen Bromide	538-71-6	50	50	NA	NA	50
Doxorubicin	25316-40-9	NA	50	NA	NA	50
Dronedarone HCl (Multaq)	141625-93-6	50	50	NA	NA	50
Epirubicin HCl	56390-09-1	NA	50	NA	NA	50
Gatifloxacin	112811-59-3	NA	NA	50	50	50
Idarubicin	57852-57-0	NA	50	NA	NA	50
Moxifloxacin	186826-86-8	NA	NA	50	NA	50
Otilonium Bromide	26095-59-0	50	50	NA	NA	50
Penfluridol	26864-56-2	NA	50	NA	NA	50
Tamoxifen	54965-24-1	50	50	NA	NA	50
Terfenadine	50679-08-8	NA	50	NA	NA	50

Table S2:

9-Aminoacridine	90-45-9
Abitrexate	59-05-2
Acipimox	51037-30-0
Adrucil	51-21-8
Cinacalcet	364782-34-3
Amoxicillin	26787-78-0
Amoxicillin	34642-77-8
Azlocillin	37091-65-9
Bacitracin	1405-87-4
Bazedoxifene	198480-56-7
Benzbromarone	3562-84-3
Bifonazole	60628-96-8
Brinzolamide	138890-62-7
Caspofungin	179463-17-3
Celecoxib	169590-42-5
Chlorprothixene	113-59-7
Clorsulon	60200-06-8
Clotrimazole	23593-75-1
Crizotinib	877399-52-5
Curcumin	458-37-7
Diclazuril	101831-37-2
Diethylstilbestrol	56-53-1
Duloxetine	136434-34-9
Econazole	24169-02-6
Elvitegravir	697761-98-1
Ethoxzolamide	452-35-7
Famotidine	76824-35-6
Fosaprepitant	265121-04-8
Isoconazole	24168-96-5
Ivacaftor	873054-44-5
Licofelone	156897-06-2
Miconazole	22916-47-8
Miconazole	22832-87-7
Mitoxantrone	70476-82-3
Nadifloxacin	124858-35-1
Nebivolol	152520-56-4
Nifuroxazide	965-52-6
Oxethazaine	126-27-2
Oxytetracycline	6153-64-6
Pitavastatin	147526-32-7
Ponatinib	943319-70-8
Pralatrexate	146464-95-1
Prochlorperazine	84-02-6
Pyrithione	13463-41-7
Sertraline	79559-97-0
Sulconazole	82382-23-8
Telmisartan	144701-48-4

Teriflunomide	108605-62-5
Tetracycline	64-75-5
Ticagrelor	274693-27-5
Tioconazole	65899-73-2
Tolfenamic	13710-19-5
Trifluoperazine	440-17-5
Trifluridine	70-00-8
Trimethoprim	738-70-5
Verteporfin	129497-78-5
Zafirlukast	107753-78-6
Axitinib	319460-85-0
Lapatinib	388082-77-7
Vandetanib	443913-73-3
Anastrozole	120511-73-1
Cladribine	4291-63-8
Bendamustine	3543-75-7
Etoposide	33419-42-0
Vincristine	2068-78-2
Posaconazole	171228-49-2
Altretamine	645-05-6
Camptothecin	7689-03-4
Megestrol	595-33-5
Felbamate	25451-15-4
Ivermectin	70288-86-7
Doripenem	364622-82-2
Mosapride	112885-42-4
Stavudine	3056-17-5
Alfuzosin	81403-68-1
Tizanidine	64461-82-1
Atazanavir	229975-97-7
Alprostadil	745-65-3
Pimobendan	74150-27-9
Olmesartan	144689-63-4
Silodosin	160970-54-7
Ethinyl	57-63-6
Amphotericin	1397-89-3
Ketorolac	74103-07-4
Enalaprilat	84680-54-6
Aminoglutethimide	125-84-8
Sulfanilamide	63-74-1
Desonide	638-94-8
Deferasirox	201530-41-8
Indomethacin	53-86-1
Mesna	19767-45-4
Esomeprazole	161973-10-0
Suprofen	40828-46-4
Oxytetracycline	79-57-2
Betaxolol	63659-19-8

Albendazole	54029-12-8
Afatinib	439081-18-2
Lenalidomide	191732-72-6
Vorinostat	149647-78-9
Docetaxel	114977-28-5
Aprepitant	170729-80-3
Decitabine	2353-33-5
Nelarabine	121032-29-9
Evista	82640-04-8
Agomelatine	138112-76-2
Prasugrel	150322-43-3
Amisulpride	71675-85-9
Carmofur	61422-45-5
Mercaptopurine	50-44-2
Fluconazole	86386-73-4
Ketoconazole	65277-42-1
Gestodene	60282-87-3
Nafamostat	82956-11-4
Tenofovir	202138-50-9
Clopidogrel	120202-66-6
Topiramate	97240-79-4
Marbofloxacin	115550-35-1
Fludarabine	21679-14-1
Tadalafil	171596-29-5
Pomalidomide	19171-19-8
Cefdinir	91832-40-5
Riluzole	1744-22-5
Naproxen	26159-34-2
Ibuprofen	15687-27-1
Adenosine	58-61-7
Dofetilide	115256-11-6
Aminophylline	317-34-0
Betamethasone	5593-20-4
Didanosine	69655-05-6
Piroxicam	36322-90-4
Terbinafine	91161-71-6
Methocarbamol	532-03-6
Niacin	59-67-6
Pyrazinamide	98-96-4
Simvastatin	79902-63-9
Acyclovir	59277-89-3
Proparacaine	5875-06-9
Chloroxine	773-76-2
Pefloxacin	70458-95-6
Bortezomib	179324-69-7
Nilotinib	641571-10-0
Masitinib	790299-79-5
Paclitaxel	33069-62-4

Bicalutamide	90357-06-5
Dutasteride	164656-23-9
Bleomycin	9041-93-4
Leflunomide	75706-12-6
Ramelteon	196597-26-9
Aniracetam	72432-10-1
Cetirizine	83881-52-1
Streptozotocin	18883-66-4
Flumazenil	78755-81-4
Lansoprazole	103577-45-3
Drospirenone	67392-87-4
Omeprazole	73590-58-6
Tenofovir	147127-20-6
Ranolazine	95635-56-6
Tranilast	53902-12-8
Cyclosporine	79217-60-0
Tazarotene	118292-40-3
Risperidone	106266-06-2
Nitazoxanide	55981-09-4
Amprenavir	161814-49-9
Zolmitriptan	139264-17-8
Isradipine	75695-93-1
Amorolfine	78613-38-4
Betapar	1247-42-3
Divalproex	76584-70-8
Levonorgestrel	797-63-7
Prednisolone	50-24-8
Nimodipine	66085-59-4
Quetiapine	111974-72-2
Ethionamide	536-33-4
Ramipril	87333-19-5
Nifedipine	21829-25-4
Pranlukast	103177-37-3
Lomustine	13010-47-4
Metoprolol	392-17-7
Bosutinib	380843-75-4
Pazopanib	635702-64-6
Capecitabine	154361-50-9
Fulvestrant	129453-61-8
Melatonin	73-31-4
Clofarabine	123318-82-1
Fludarabine	75607-67-9
Enzalutamide	915087-33-1
Artemisinin	63968-64-9
Cilnidipine	132203-70-4
Dexamethasone	50-02-2
Fluoxetine	56296-78-7
Levetiracetam	102767-28-2

Ruxolitinib	941678-49-5
Ondansetron	99614-01-4
Tigecycline	220620-09-7
Repaglinide	135062-02-1
Venlafaxine	99300-78-4
Calcitriol	32222-06-3
Betamethasone	378-44-9
Natamycin	7681-93-8
Sulfasalazine	599-79-1
Rizatriptan	145202-66-0
Sulfameter	651-06-9
Triamcinolone	76-25-5
Albendazole	54965-21-8
Telbivudine	3424-98-4
Estrone	53-16-7
Chloramphenicol	56-75-7
Betamethasone	2152-44-5
Emtricitabine	143491-57-0
Glipizide	29094-61-9
Gemfibrozil	25812-30-0
Nisoldipine	63675-72-9
Fenofibrate	49562-28-9
Amiloride	2016-88-8
Oxfendazole	53716-50-0
Chenodeoxycholic	474-25-9
Dasatinib	302962-49-8
Rapamycin	53123-88-9
Vismodegib	879085-55-9
Cisplatin	15663-27-1
Thalidomide	50-35-1
Bisoprolol	104344-23-2
Dacarbazine	4342-03-4
Topotecan	119413-54-6
Dienogest	65928-58-7
Asenapine	65576-45-6
Cilostazol	73963-72-1
Doxazosin	77883-43-3
Fluvoxamine	61718-82-9
Lidocaine	137-58-6
Isotretinoin	4759-48-2
Oxcarbazepine	28721-07-5
Trilostane	13647-35-3
Rolipram	61413-54-5
Voriconazole	137234-62-9
Doxercalciferol	54573-75-0
Mycophenolate	128794-94-5
Telaprevir	402957-28-2
Candesartan	139481-59-7

Pyridostigmine	101-26-8
Prilocaine	721-50-6
Orlistat	96829-58-2
Chlorothiazide	58-94-6
Monobenzone	103-16-2
Flucytosine	2022-85-7
Flurbiprofen	51543-39-6
Praziquantel	55268-74-1
Progesterone	57-83-0
Glyburide	10238-21-8
Indapamide	26807-65-8
Thiabendazole	148-79-8
Oxybutynin	5633-20-5
Beta Carotene	7235-40-7
Azacitidine	320-67-2
Ranolazine	95635-55-5
Amlodipine	111470-99-6
Carvedilol	72956-09-3
Cimetidine	51481-61-9
Diltiazem	33286-22-5
Erlotinib	183319-69-9
Sorafenib	475207-59-1
Cabozantinib	849217-68-1
Valproic	1069-66-5
Exemestane	107868-30-4
Dexrazoxane	149003-01-0
2-Methoxyestradiol	362-07-2
Entecavir	209216-23-9
Vemurafenib	918504-65-1
Benazepril	86541-74-4
Floxuridine	50-91-9
Edaravone	89-25-8
Loratadine	79794-75-5
Lopinavir	192725-17-0
Pizotifen	5189-11-7
Vecuronium	50700-72-6
Sildenafil	171599-83-0
Zileuton	111406-87-2
Alfacalcidol	41294-56-8
Cephalexin	15686-71-2
Saxagliptin	361442-04-8
Apixaban	503612-47-3
Methimazole	60-56-0
Darunavir	635728-49-3
Allopurinol	315-30-0
Ursodiol	128-13-2
Tretinoin	302-79-4
Trichlormethiazide	133-67-5

Disulfiram	97-77-8
Busulfan	55-98-1
Lamivudine	134678-17-4
Adefovir	142340-99-6
Mitotane	53-19-0
Guaifenesin	93-14-1
Enoxacin	74011-58-8
Cefditoren	117467-28-4
Vidarabine	5536-17-4
Ranitidine	66357-59-3
Chlorpheniramine	113-92-8
Atracurium	64228-81-5
Clemastine	14976-57-9
Diphenhydramine	147-24-0
Gefitinib	184475-35-2
Sunitinib	341031-54-7
Everolimus	159351-69-6
Regorafenib	755037-03-7
Finasteride	98319-26-7
Letrozole	112809-51-5
Nepafenac	78281-72-8
Acarbose	56180-94-0
Budesonide	51333-22-3
Ftorafur	17902-23-7
Etodolac	41340-25-4
Genistein	446-72-0
Losartan	124750-99-8
Meropenem	96036-03-2
Resveratrol	501-36-0
Bimatoprost	155206-00-1
Sumatriptan	103628-48-4
Ziprasidone	122883-93-6
Iloperidone	133454-47-4
Dyphylline	479-18-5
Febuxostat	144060-53-7
Reserpine	50-55-5
Metolazone	17560-51-9
Prednisone	53-03-2
Nitrofurazone	59-87-0
Phenylbutazone	50-33-9
Loteprednol	82034-46-6
Mesalamine	89-57-6
Carbamazepine	298-46-4
Hydrochlorothiazide	58-93-5
Zalcitabine	7481-89-2
Methylprednisolone	83-43-2
Sulfadiazine	68-35-9
Acadesine	2627-69-2

Fenoprofen	34597-40-5
Butoconazole	64872-77-1
Dapoxetine	129938-20-1
Imatinib	220127-57-1
Temsirolimus	162635-04-3
Malotilate	59937-28-9
Ritonavir	155213-67-5
Irinotecan	97682-44-5
Oxaliplatin	61825-94-3
Methazolastone	85622-93-1
Rufinamide	106308-44-5
Adapalene	106685-40-9
Bumetanide	28395-03-1
Ifosfamide	3778-73-2
Etomidate	33125-97-2
Glimepiride	93479-97-1
Acitretin	55079-83-9
Mianserin	21535-47-7
Rocuronium	119302-91-9
Tianeptine	30123-17-2
Zonisamide	68291-97-4
Naratriptan	143388-64-1
Aztreonam	78110-38-0
Furosemide	54-31-9
Cefoperazone	62893-19-0
Acetylcysteine	616-91-1
Erythromycin	114-07-8
Ketoprofen	22071-15-4
Ezetimibe	163222-33-1
Aminocaproic	60-32-2
Ipratropium	22254-24-6
Hydrocortisone	50-23-7
Estradiol	50-28-2
Azathioprine	446-86-6
Meloxicam	71125-38-7
Nevirapine	129618-40-2
Teniposide	29767-20-2
Acetylcholine	60-31-1
Erdosteine	84611-23-4
Azithromycin	83905-01-5
Daidzein	486-66-8
Valaciclovir	124832-27-5
Ganciclovir	82410-32-0
Carbidopa	28860-95-9
Diclofenac	15307-79-6
Pregnenolone	145-13-1
Triamcinolone	124-94-7
Sulfamethizole	144-82-1

Nicorandil	65141-46-0
Propylthiouracil	51-52-5
Pramipexole	191217-81-9
Ginkgolide	15291-75-5
Lornoxicam	70374-39-9
Terazosin	70024-40-7
Argatroban	74863-84-6
Ambrisentan	177036-94-1
Imidapril	89371-37-9
Roflumilast	162401-32-3
Irinotecan	136572-09-3
Nalidixic	389-08-2
Genipin	6902-77-8
Bethanechol	590-63-6
Famciclovir	104227-87-4
Manidipine	89226-50-6
Olanzapine	132539-06-1
Racecadotril	81110-73-8
Vardenafil	330808-88-3
Acetanilide	103-84-4
Sulbactam	69388-84-7
Dimethyl Fumarate	624-49-7
Acemetacin	53164-05-9
Cobicistat	1004316-88-4
Aspirin	50-78-2
Fenoprofen	71720-56-4
Rofecoxib	162011-90-7
Medetomidine	86347-15-1
Etravirine	269055-15-4
Vitamin C	50-81-7
Sulfamethazine	57-68-1
Roxatidine	93793-83-0
Valsartan	137862-53-4
Avobenzone	70356-09-1
Sulfamethoxazole	723-46-6
Nystatin	1400-61-9
Sulbactam	68373-14-8
Fluticasone	80474-14-2
Suplatast	94055-76-2
Phentolamine	65-28-1
Captopril	62571-86-2
Bromhexine	611-75-6
Mecarbinate	15574-49-9
Trimebutine	39133-31-8
Bexarotene	153559-49-0
Lapatinib	231277-92-2
Dextrose	50-99-7
Apatinib	811803-05-1

Ammonium	1407-03-0
Geniposidic	27741-01-1
Chlorpromazine	69-09-0
Fenbendazole	43210-67-9
Milrinone	78415-72-2
Olopatadine	140462-76-6
Ribavirin	36791-04-5
Xylazine	23076-35-9
Ciclopirox	29342-05-0
Clomipramine	17321-77-6
Azelastine	79307-93-0
Cloxacillin	7081-44-9
Xylometazoline	1218-35-5
Miglitol	72432-03-2
Tioxolone	4991-65-5
Arecoline	300-08-3
Carbazochrome	51460-26-5
Niflumic	4394-00-7
Linagliptin	668270-12-0
Cinepazide	26328-04-1
Diclofenac Potassium	15307-81-0
Ulipristal	159811-51-5
Sulfathiazole	72-14-0
Sodium	54-21-7
Protionamide	14222-60-7
Dipyridamole	58-32-2
Amlodipine	88150-42-9
Sulfisoxazole	127-69-5
Isoniazid	54-85-3
Meglumine	6284-40-8
Lacidipine	103890-78-4
Mirtazapine	85650-52-8
Uridine	58-96-8
Nimesulide	51803-78-2
Lovastatin	75330-75-5
Rosiglitazone	302543-62-0
Ivabradine	148849-67-6
Temocapril	110221-44-8
Cisatracurium	96946-42-8
Xylose	25990-60-7
Dabigatran	211915-06-9
TAME	901-47-3
D-Mannitol	69-65-8
Tolbutamide	64-77-7
Clindamycin	21462-39-5
Fluocinolone	67-73-2
Oxymetazoline	2315-02-8
Rosiglitazone	155141-29-0

Maprotiline	10347-81-6
Dopamine	62-31-7
Phenformin	834-28-6
5-Aminolevulinic	5451-09-2
Phenacetin	62-44-2
Pioglitazone	111025-46-8
Dehydroepiandrosterone	53-43-0
Noradrenaline	108341-18-0
Rivaroxaban	366789-02-8
Ciclopirox	41621-49-2
Bindarit	130641-38-2
Azilsartan	147403-03-0
Diclofenac	78213-16-8
Indacaterol	753498-25-8
Oxybutynin	1508-65-2
Methylthiouracil	56-04-2
Idoxuridine	54-42-2
Hydroxyurea	127-07-1
Metronidazole	443-48-1
Crystal violet	548-62-9
Levofloxacin	100986-85-4
Pranoprofen	52549-17-4
Aripiprazole	129722-12-9
Benidipine	91599-74-5
Flunarizine	30484-77-6
Dyclonine	536-43-6
Cytidine	65-46-3
Tiopronin	1953-02-2
Atorvastatin	134523-03-8
Rivastigmine	129101-54-8
Gabexate	56974-61-9
Mestranol	72-33-3
Tebipenem	161715-24-8
Eltrombopag	496775-62-3
Sorbitol	50-70-4
Levosimendan	141505-33-1
Clonidine	4205-91-8
Gallamine	65-29-2
Moroxydine	3160-91-6
Ozagrel	82571-53-7
Roxithromycin	80214-83-1
Naphazoline	550-99-2
Ritodrine	23239-51-2
Ceftiofur	103980-44-5
Clarithromycin	81103-11-9
Isoprenaline	51-30-9
Zidovudine	30516-87-1
Tolvaptan	150683-30-0

Idebenone	58186-27-9
Ibrutinib	936563-96-1
Paroxetine	78246-49-8
Rimonabant	168273-06-1
Vildagliptin	274901-16-5
Naloxone	357-08-4
2-Thiouracil	141-90-2
Ornidazole	16773-42-5
Methenamine	100-97-0
Sparfloxacin	110871-86-8
Potassium	7681-11-0
Flutamide	13311-84-7
Haloperidol	52-86-8
Enalapril	76095-16-4
Sulphadimethoxine	122-11-2
Methscopolamine	155-41-9
Maraviroc	376348-65-1
Formoterol	43229-80-7
Fenticonazole	73151-29-8
Memantine	41100-52-1
Orphenadrine	4682-36-4
Dexmedetomidine	145108-58-3
Rasagiline	161735-79-1
Conivaptan	168626-94-6
Naftopidil	57149-07-2
Rosuvastatin	147098-20-2
Esomeprazole	161796-78-7
Cephalomannine	71610-00-9
Amantadine	665-66-7
Clozapine	5786-21-0
Imatinib	152459-95-5
Mycophenolic	24280-93-1
Pancuronium	15500-66-0
Scopolamine	114-49-8
Epinephrine	51-42-3
Scopine	498-45-3
Rosiglitazone	122320-73-4
Medroxyprogesterone	71-58-9
Quinapril	82586-55-8
Pramiracetam	68497-62-1
Mifepristone	84371-65-3
Nilvadipine	75530-68-6
Zanamivir	139110-80-8
Cabazitaxel	183133-96-2
Solifenacin	242478-38-2
Moguisteine	119637-67-1
Dexamethasone	1177-87-3
Milnacipran	101152-94-7

Felodipine	72509-76-3
Tropisetron	105826-92-4
Fluvastatin	93957-55-2
Phenindione	83-12-5
Menadione	58-27-5
Rimantadine	13392-28-4
Amiodarone	19774-82-4
Raltegravir	518048-05-0
Chlormezanone	80-77-3
Rebamipide	90098-04-7
Cyproheptadine	969-33-5
Gimeracil	103766-25-2
Lafutidine	118288-08-7
Moexipril	82586-52-5
Betaxolol	659-18-7
Naltrexone	16676-29-2
Ibutilide	122647-32-9
S-(+)-Rolipram	85416-73-5
Aliskiren	173334-58-2
Fesoterodine	286930-03-8
10-Deacetylbaccatin-III	32981-86-5
Amfebutamone	31677-93-7
Pramipexole	104632-26-0
Itraconazole	84625-61-6
Nateglinide	105816-04-4
Phenoxybenzamine	63-92-3
Sotalol	959-24-0
L-Adrenaline	51-43-4
Tiotropium	139404-48-1
Terbinafine	78628-80-5
Phenylephrine	61-76-7
Clindamycin	25507-04-4
Buflomedil	35543-24-9
Dabrafenib	1195765-45-7
Zaltoprofen	74711-43-6
Bufexamac	2438-72-4
Pravastatin	81131-70-6
Azelnidipine	123524-52-7
Dexmedetomidine	113775-47-6
Darifenacin	133099-07-7
Deflazacort	14484-47-0
Nicotinamide	98-92-0
Alibendol	26750-81-2
Methoxsalen	298-81-7
Primidone	125-33-7
Adenine	2922-28-3
Pyrimethamine	58-14-0
Ketotifen	34580-14-8

Epalrestat	82159-09-9
Doxifluridine	3094-09-5
Cyclophosphamide	6055-19-2
Moxonidine	75438-57-2
Cleviprex	167221-71-8
Detomidine	90038-01-0
Levosulpiride	23672-07-3
Probucol	23288-49-5
Desmethyl Erlotinib	183320-51-6
Artemether	71963-77-4
Paeoniflorin	23180-57-6
Benserazide	14919-77-8
Domperidone	57808-66-9
Lincomycin	859-18-7
Nitrendipine	39562-70-4
Propafenone	34183-22-7
Spectinomycin	21736-83-4
Phenytoin	630-93-3
Trospium	10405-02-4
Cortisone	50-04-4
Prednisolone	52-21-1
Clobetasol	25122-46-7
L-Thyroxine	51-48-9
Fluocinonide	356-12-7
Clindamycin	18323-44-9
Pazopanib	444731-52-6
Lamotrigine	84057-84-1
Bepotastine	190786-44-8
Alverine	5560-59-8
Beclomethasone	5534-09-8
Pidotimod	121808-62-6
Biotin	58-85-5
Tripelennamine	154-69-8
Nizatidine	76963-41-2
Vitamin	68-19-9
Tropicamide	1508-75-4
Irsogladine	57381-26-7
Nefiracetam	77191-36-7
Mometasone	83919-23-7
Sulindac	38194-50-2
Urapidil	64887-14-5
Aspartame	22839-47-0
Pioglitazone	112529-15-4
Tolnaftate	2398-96-1
Ozagrel	78712-43-3
Adiphenine	50-42-0
Almotriptan	181183-52-8
Flunixin	42461-84-7

Arbidol	131707-23-8
Atropine	5908-99-6
DAPT	208255-80-5
DL-Carnitine	461-05-2
Geniposide	24512-63-8
Bupivacaine	18010-40-7
Estriol	50-27-1
Loperamide	34552-83-5
Quinine	6119-47-7
Tenoxicam	59804-37-4
Phenytoin	57-41-0
Secnidazole	3366-95-8
Tolterodine	124937-52-6
Amiloride	17440-83-4
Tetracaine	136-47-0
Brompheniramine	980-71-2
Gliclazide	21187-98-4
Lonidamine	50264-69-2
Carfilzomib	868540-17-4
PMSF	329-98-6
Azilsartan	863031-21-4
Atovaquone	95233-18-4
Pyridoxine	58-56-0
Sulfamerazine	127-79-7
Entacapone	130929-57-6
Estradiol	979-32-8
Benztropine	132-17-2
Carbenicillin	4800-94-6
Azacyclonol	115-46-8
Moclobemide	71320-77-9
Desloratadine	100643-71-8
Probenecid	57-66-9
Vitamin	50-14-6
toltrazuril	69004-03-1
Vitamin	67-97-0
Lomerizine	101477-54-7
Droperidol	548-73-2
Deoxyarbutin	53936-56-4
Amfenac	61618-27-7
Doxofylline	69975-86-6
1-Hexadecanol	36653-82-4
Penciclovir	39809-25-1
Chlorquinaldol	72-80-0
Benzocaine	94-09-7
Pilocarpine	54-71-7
Meclofenamate	6385-02-0
Diphenylpyraline	132-18-3
Metaraminol	33402-03-8

Procyclidine	1508-76-5
Noscapine	912-60-7
Acetarsone	97-44-9
Oxeladin	52432-72-1
Tacrolimus	104987-11-3
Articaine	23964-57-0
Altrenogest	850-52-2
Flumequine	42835-25-6
Reboxetine	98769-84-7
Pergolide	66104-23-2
Hyoscyamine	101-31-5
Procaine	51-05-8
Doxapram	7081-53-0
Pheniramine	132-20-7
Spironolactone	52-01-7
Escitalopram	219861-08-2
Propranolol	318-98-9
Levobetaxolol	116209-55-3
Dydrogesterone	152-62-5
Clofazimine	2030-63-9
Clorprenaline	6933-90-0
Benzydamine	132-69-4
Sulfaguanidine	57-67-0
Tiratricol	51-24-1
Azaguanine-8	134-58-7
Furaltadone	3759-92-0
Montelukast	151767-02-1
Piperacillin	59703-84-3
Nithiamide	140-40-9
Deoxycorticosterone	56-47-3
Disopyramide	22059-60-5
Meticrane	1084-65-7
Ractopamine	90274-24-1
Phenothrin	26002-80-2
Mepenzolate	76-90-4
Bephenium	3818-50-6
Pasiniazid	2066-89-9
Pimecrolimus	137071-32-0
Gliquidone	33342-05-1
Ampicillin	69-52-3
Amitriptyline	549-18-8
Triflusal	322-79-2
Lithocholic	434-13-9
Cyclamic	100-88-9
Homatropine	80-49-9
Dibucaine	61-12-1
Estradiol	313-06-4
Guanabenz	23256-50-0

Mequinol	150-76-5
Loxapine	27833-64-3
Dexlansoprazole	138530-94-6
Dicloxacillin	13412-64-1
Carprofen	53716-49-7
Ethamsylate	2624-44-4
Chlorpropamide	94-20-2
Trometamol	77-86-1
Broxyquinoline	521-74-4
Isosorbide	652-67-5
Dirithromycin	62013-04-1
Mevastatin	73573-88-3
Zoxazolamine	61-80-3
Serotonin	153-98-0
Moxalactam	64953-12-4
Phthalylsulfacetamide	131-69-1
Aceclidine	6109-70-2
Brucine	652154-10-4
Butenafine	101827-46-7
Anagrelide	58579-51-4
Adrenalone	62-13-5
Ethambutol	1070-11-7
Ouabain	630-60-4
Homatropine	51-56-9
Methazolamide	554-57-4
Bisacodyl	30652-11-0
Methyclothiazide	135-07-9
tinidazole	19387-91-8
Mefenamic	61-68-7
Flumethasone	2135-17-3
Esmolol	81161-17-3
Triclabendazole	68786-66-3
Dropropizine	17692-31-8
Chlorzoxazone	95-25-0
Cyromazine	66215-27-8
Uracil	66-22-8
Salicylanilide	87-17-2
Ethacridine	6402-23-9
Dibenzothiophene	132-65-0
Sucralose	56038-13-2
Mexiletine	5370-01-4
Phenazopyridine	136-40-3
Sodium	134-03-2
Anisotropine	80-50-2
Famprofazone	22881-35-2
Nalmefene	58895-64-0
Tacrine	1684-40-8
Carbenoxolone	7421-40-1

Imipramine	113-52-0
Camylofin	54-30-8
Procodazole	23249-97-0
Mepivacaine	1722-62-9
Antipyrine	60-80-0
Azatadine	3978-86-7
Catharanthine	2468-21-5
Pentamidine	140-64-7
Allylthiourea	109-57-9
Hydroxyzine	2192-20-3
norethindrone	68-22-4
Carbimazole	22232-54-8
Ropivacaine	98717-15-8
Guanidine	50-01-1
Halobetasol	66852-54-8
Voglibose	83480-29-9
Isovaleramide	541-46-8
Amprolium	137-88-2
Bezafibrate	41859-67-0
Climbazole	38083-17-9
Sasapyrine	552-94-3
Bemegride	64-65-3
Cysteamine	156-57-0
Primaquine	63-45-6
Vinorelbine	125317-39-7
Benzthiazide	91-33-8
Isoetharine	7279-75-6
Nialamide	51-12-7
Pimozide	2062-78-4
Nicotine	65-31-6
Proadifen	62-68-0
Clofoctol	37693-01-9
Sodium	94-16-6
Ethynodiol	297-76-7
Atomoxetine	82248-59-7
(+,-)-Octopamine	770-05-8
Meptazinol	59263-76-2
Mirabegron	223673-61-8
Avanafil	330784-47-9
Flavoxate	3717-88-2
olsalazine	6054-98-4
Bextra	181695-72-7
Sodium Nitroprusside	14402-89-2
Decamethonium	541-22-0
triamterene	396-01-0
Fenspiride	5053-08-7
Eprosartan	144143-96-4
Penicillin	69-57-8

Coumarin	91-64-5
Mezlocillin	42057-22-7
Cyclandelate	456-59-7
Aminothiazole	96-50-4
Clofibric	882-09-7
Liothyronine	55-06-1
Fluorometholone	3801-06-7
Cepharanthine	481-49-2
Rotigotine	99755-59-6
Calcium	17140-60-2
Mepiroxol	6968-72-5
Carbachol	51-83-2
Pridinol	6856-31-1
Pyrilamine	59-33-6
Diperodon	537-12-2
Trimipramine	521-78-8
Sertaconazole	99592-39-9
Betahistine	5579-84-0
Ropinirole	91374-20-8
Fexofenadine	153439-40-8
Acebutolol	34381-68-5
Sodium	10040-45-6
Aclidinium	320345-99-1
nafcillin	7177-50-6
valganciclovir	175865-59-5
Erythromycin	1264-62-6
Aminosalicylate	133-10-8
sulfacetamide	127-56-0
Pramoxine	637-58-1
Diminazene	908-54-3
Tilmicosin	108050-54-0
Azithromycin	117772-70-0
Benzoic	65-85-0
Choline	67-48-1
Nicardipine	54527-84-3
Cinchophen	132-60-5
Antazoline	2508-72-7
Chromocarb	4940-39-0
Azaperone	1649-18-9
Oxybuprocaine	5987-82-6
Bergapten	484-20-8
Nelfinavir	159989-65-8
Carbadox	6804-07-5
Mesoridazine	32672-69-8
Pentoxifylline	6493-05-6
Tolmetin	64490-92-2
Triflupromazine	1098-60-8
Difloxacin	91296-86-5

Isoxicam	34552-84-6
Vinblastine	143-67-9
Tylosin	74610-55-2
Amidopyrine	58-15-1
Ampiroxicam	99464-64-9
Tolcapone	134308-13-7
Diphemanil	62-97-5
tetrahydrozoline	522-48-5
Nabumetone	42924-53-8
Ronidazole	7681-76-7
Sodium	7632-00-0
Spiramycin	8025-81-8
Difluprednate	23674-86-4
Troxipide	30751-05-4
Ampicillin	7177-48-2
Betamipron	3440-28-6
Tolperisone	3644-61-9
Chlorocresol	59-50-7
Bosentan	147536-97-8
Oxaprozin	21256-18-8
Doxylamine	562-10-7
Ospemifene	128607-22-7
Ceftazidime	78439-06-2
Metaproterenol	5874-97-5
Piromidic	19562-30-2
Glafenine	65513-72-6
Dicyclomine	67-92-5
Fosfomycin	78964-85-9
Nifenazone	2139-47-1
Tofacitinib	540737-29-9
